# Auditing cognitive drift in AI-driven recommendation: a responsible AI methods protocol with a health case demonstration

**DOI:** 10.3389/fnins.2025.1697053

**Published:** 2025-12-08

**Authors:** Zhuowei Li, Congqian Zhu

**Affiliations:** 1 Universitat Zurich Wirtschaftswissenschaftliche Fakultat, Zürich, Switzerland; 2 Artificial Intelligence Research Institute, Shenzhen University of Advanced Technology, Shenzhen, China

**Keywords:** cognitive drift, algorithmic recommendation, explainable measurement, platform governance, responsible artificial intelligence, health communication

## Abstract

We propose a protocol to detect and track cognitive drift caused by algorithmic curation. We confirm that the protocol is interpretable, sensitive, reproducible, and portable across domains. It is especially suited to cognitive and neurocognitive research. We build a Cognitive Drift Index (CDI), confirm its three dimensions, and use a small calibration run to set reasonable ranges. We then map CDI to governance action bands. Using health short videos as a case, we estimate path effects with a weighted least squares ANOVA and test robustness. The steps lead to the same pattern, which supports the protocol’s design and practical use. We show each component alongside the composite and sort totals into action bands so practitioners and responsible-AI teams can choose proportionate actions. We also consider using the method, as a conceptual tool, with eye-tracking or EEG to enable multimodal validation.

## 1 Introduction

In contemporary society, digital platforms have become one of the primary channels through which people receive information. They determine what appears, how long people attend to it, and which cues are noticed, creating steady day-to-day guidance. Over time, that steer gradually resets cognitive baselines. We call this “cognitive drift,” namely the effect that accumulates through long-term, low-frequency contact with information, in which subtle changes in evaluative attention and interpretive focus build up. In contrast to classic attitude-change theories that emphasize prior beliefs and explicit persuasion, we examine how the architecture of exposure paths shapes cognition. AI-driven curation sets those paths and quietly reweights signals—raising some and lowering others—so different effects emerge even when users are not actively seeking change (Tufekci, 2015; Covington et al., 2016; Bucher, 2018).

This account is conceptually close to several familiar ideas but differs in important ways. It differs from selective exposure and “filter-bubble” views: the main driver is not people’s own choices but the exposure path shaped by recommendation algorithms (Nguyen et al., 2014; Pariser, 2011). Because we focus on a human-level phenomenon—the gradual reweighting of attention and evaluation within exposure architectures—it also departs from macro explanations centered on datafied political economy (Zuboff, 2023; van Dijck et al., 2018). On the supply side, creators tweak titles, tags, and formats to match ranking systems. That shrinks the range of content and makes user paths look more alike (Gillespie, 2010). For cognitive impact, we examine how emotionally charged and repetitious content layouts are widely taken up by audiences, creating conditions under which algorithms and AI can shift cognition (Senft and Baym, 2015; Lupton, 2017; Nagler and LoRusso, 2017). This also includes the human labor of content moderation (Roberts, 2019).

To make such slow change observable and auditable, this article presents a methods protocol for detecting and monitoring cognitive drift with an emphasis on interpretability, sensitivity, and reproducibility. The concept of cognitive drift that we propose averages, with equal weight, three dimensions: emotional drift (valence change between rounds), tag homogeneity (similarity of tags within a round), and selective salience (the degree to which attention concentrates across categories). Each dimension is readable on its own; taken together, they reveal the force that plays a leading role and help to determine whether such cognitive change is affect-driven or structure-dominated. These choices rest on established evidence: emotions steer attention (Lang et al., 2013; Hajcak et al., 2010), and set-based similarity offers a simple, robust way to track label convergence (Real and Vargas, 1996).

We decompose CDI into update amplitude, direction (polarity), and conflicting cue weights, corresponding to prediction-error-driven belief updates, value/valence-encoding-guided orientation shifts, and conflict-monitoring-controlled weight reassortments, respectively. This is a mechanism-anchored tripartite approach rather than for operational convenience (Friston, 2010; Botvinick et al., 2001). The update amplitude is derived from the encoding of prediction errors by the dopaminergic medial frontal lobe pathway, which determines to what extent new evidence should be updated relative to the prior (Schultz et al., 1997; Friston, 2009). Direction (polarity) is regulated by the value/valence encoding and familiarity in the vmPFC/ventral striatum, resulting in a systematic shift towards convergence or divergence during integration (Levy and Glimcher, 2012; Liu et al., 2023a,b).

The weight of conflicting cues corresponds to the conflict-monitoring control recruitment process led by the ACC. When evidence conflicts, the control network upregulates to reconfigure attention and decision weights, and the integration cost increases accordingly (Botvinick et al., 2001; Seeley et al., 2007).

The above-mentioned three dimensions are co-regulated by attention/saliency gating: the top-down attention network determines when/what is elevated as the control target, and eye-movement and EEG evidence also shows that gaze patterns and consistency systematically change the amplitude and direction of subsequent updates (Corbetta and Shulman, 2002; Liu et al., 2025a,b,c).

Based on this mechanism anchoring, the two-step workflow described below transparently operationalizes three-dimensional concepts and conducts auditable path effect tests (Liu et al., 2025a,b,c; Friston, 2010).

To help neuroscience readers quickly align terminology, we map the three components to typical neural processes and clarify the scope of inference for this study: EB reflects the update direction and step size driven by prediction error; TC characterizes the aggregation strength of representational/semantic structure (with label homogeneity as a checkable representation); and SS reflects concentration under attention and salience gating. For the sake of auditability and reproducibility, we use behavioral signals such as clicks and dwell time as engineering proxies for interpretable monitoring at the group/round level. Therefore, this study does not claim causal effects at the individual level, and fine-grained mechanisms at the individual level need to be further validated in subsequent studies using physiological recordings. Based on this definition, a two-step workflow is provided below.

The workflow has two parts. First, a small calibration run sets sensible ranges for each component and sets bands on the CDI score for Responsible-AI use. Think simple actions: a “why am I seeing this?” prompt, light diversity rules, or short-term affect guards. Second, we test on real data with a Type-II WLS ANOVA to see path effects. We then sanity-check the result with small weight tweaks (±10%) and an OLS model using HC3 errors. The goal is use, not flash: you should be able to run it, explain it, and stand behind it (Yeung, 2018; Metcalf et al., 2021; Raji et al., 2020; Mittelstadt, 2019).

In practice, those bands point to right-sized responses and do not lock the method to one domain. We walk through a health short-video case to show the steps, but the same setup fits news, e-commerce and product discovery, civic and public-health portals, and education. It also pairs well with neurocognitive checks: eye-tracking and pupillometry can track attention alongside SS (Holmqvist et al., 2011; Mathôt and Vilotijević, 2023). Neuroimaging can relate representational or network change to exposure architecture in designs that mirror our paths (Kriegeskorte and Kievit, 2013; Bressler and Menon, 2010; Redcay and Moraczewski, 2020). Our aim is simple: an interpretable, sensitive, reproducible tool people can use—and trust.

## 2 Materials and equipment

### 2.1 Data sources and inputs

While building and testing this method, we worked with health-related short videos that anyone can access. We gathered them from June to September 2023 using the platform’s search and trending pages. For each item, we kept the basic metadata—title, tags, and text description—and two coarse attention signals: clicks and dwell time in seconds. No personal identifiers were collected.

Exposure sequences are constructed under three path structures—active search, semi-recommendation, and pure recommendation—and aggregated at path × round and path × content × round levels for analysis. Seen through a path lens, what reaches a user is not accidental. Ranking and interface work together to decide what appears first, what lingers, and what quietly slips from view—shaping what feels “normal” to see (Tufekci, 2015; Bucher, 2018). At scale, recommender pipelines push discovery past the first query, often carrying users farther than they intended (Covington et al., 2016). The ad-tech and personalization stack is mostly hidden; small, behind-the-scenes changes can nudge someone onto a different track without their noticing (Eslami et al., 2018). At the systems level, platforms operate as connective infrastructure: the rules they set—and the guardrails they choose or decline—govern how information flows, who gets a hearing, and who fades into the background (van Dijck et al., 2018; Gillespie, 2018).

### 2.2 Content coding and quality control

We divided the items into four categories: popular-science content from authoritative sources; intervention advice based on practical experience; product-promotion health content; and content lacking a scientific basis or carrying an anxiety-framing. Two researchers independently coded each item. Agreement was high, and we resolved small differences through discussion.

With these source-oriented labels in place, we defined three key indicators for the subsequent analysis: the trajectory of tone across rounds, the overlap of tags within a single round, and the distribution of attention across categories. Our results again support the view that, compared with neutral material, emotionally strong and exposure-optimized posts tend to spread farther and draw more attention (Senft and Baym, 2015; Lupton, 2017). When claims are complex or contested, people also rely on affective resonance and familiar cues as quick heuristics, so the exposure structure can quietly tilt judgments (Nagler and LoRusso, 2017).

For affect, we scored each item with an in-domain Chinese valence lexicon (neutral ≈ 0.50). We ran stratified spot checks and corrected obvious misclassifications. Treating valence as a proxy is in line with prior work: emotion steers motivated attention and regulation during evaluation (Lang et al., 2013; Hajcak et al., 2010), and ERP studies show that incidental affect can shift cognition under load (Liang et al., 2021).

From these inputs we compute the three parts of the Cognitive Drift Index (CDI). Emotional drift (EB) is the round-to-round change in mean valence, smoothed with a short moving average. Tag homogeneity (TC) is the mean pairwise Jaccard similarity of tokenized tags within a round, a clear set-based way to track convergence (Real and Vargas, 1996). Selective salience (SS) shows how attention concentrates across categories, using shares from clicks (weight = 1.0) and dwell time (0.05 per second). Auditing attention shares is a standard way to spot visibility skew in curated feeds (Tufekci, 2015; Bucher, 2018).

### 2.3 Software and reproducibility resources

All processing was implemented in Python (≥3.10) using NumPy and Pandas for data wrangling, SciPy for sampling in the calibration simulation, and statsmodels for estimation and robust covariance. The pre-study simulation employs a fixed random seed (2023) for reproducibility. The replication package (released upon acceptance) includes: (i) cleaning and aggregation scripts that build the path × round and path × content × round tables; (ii) functions to compute EB, TC, SS, and the composite CDI; (iii) simulation code for calibration and threshold checks; and (iv) scripts that reproduce all figures and statistics. Robust inference options (e.g., HC3 covariance) follow established guidance on heterosexuality-consistent estimation (MacKinnon and White, 1985), and model diagnostics are reported in line with standard practice for linear models (Fox and Weisberg, 2011).

To keep the review blind, we cite only the derived affect scores. If the paper is accepted, we will post the exact affect lexicon and its SHA-256 checksum so anyone can recompute the indices. This level of disclosure matches current practice in explainable, accountable AI for human-facing work (Miller, 2019; Kaur et al., 2022; Mittelstadt, 2019; Raji et al., 2020).

### 2.4 Compute, timing, and ethics

Analyses run on a standard laptop environment; end-to-end runtime for the demonstration dataset is on the order of minutes. We stripped all identifiers before analysis, and—per platform terms—do not share raw logs. Only aggregated, non-identifiable tables are released in the replication package.

The protocol is domain-portable and, where approvals permit, can be paired with additional cognitive measurements while keeping the same index construction and reporting format. Obvious bridges include eye-tracking metrics for attention allocation (Holmqvist et al., 2011; Mathôt and Vilotijević, 2023) and representational comparisons that relate categorical structure to neural encoding (Kriegeskorte and Kievit, 2013). At the network level, salience and control systems are sensible places to look when tying exposure paths to brain activity (Bressler and Menon, 2010; Menon and Uddin, 2010). Naturalistic tasks then give us a way to track slow change in settings that look more like the real world (Redcay and Moraczewski, 2020).

## 3 Methods

### 3.1 Rationale and theoretical grounding

We treat cognitive drift as a slow, path-dependent shift in how people weigh and judge cues under incidental exposure. Our goal is simple: make this change measurable, checkable, and easy to reuse across domains. On platforms, data systems learn preferences and push content that maximizes engagement; ranked feeds make those choices stick (Tufekci, 2015; Covington et al., 2016). Interface design and feedback loops make ranking part of how information is revealed (Gillespie, 2018). Creators then adapt. They tune titles, tags, and formats to please the ranking rules, which shrinks repertoires and lowers diversity (Nguyen et al., 2014). Whole creator ecosystems chase ranking signals and audience capture, locking in path-dependent exposure (Abidin, 2018). When content spreads across platforms, sameness and bias can grow, especially in health topics (Zhou et al., 2018; van Dijck et al., 2018; Noble, 2018).

**Figure 1:**
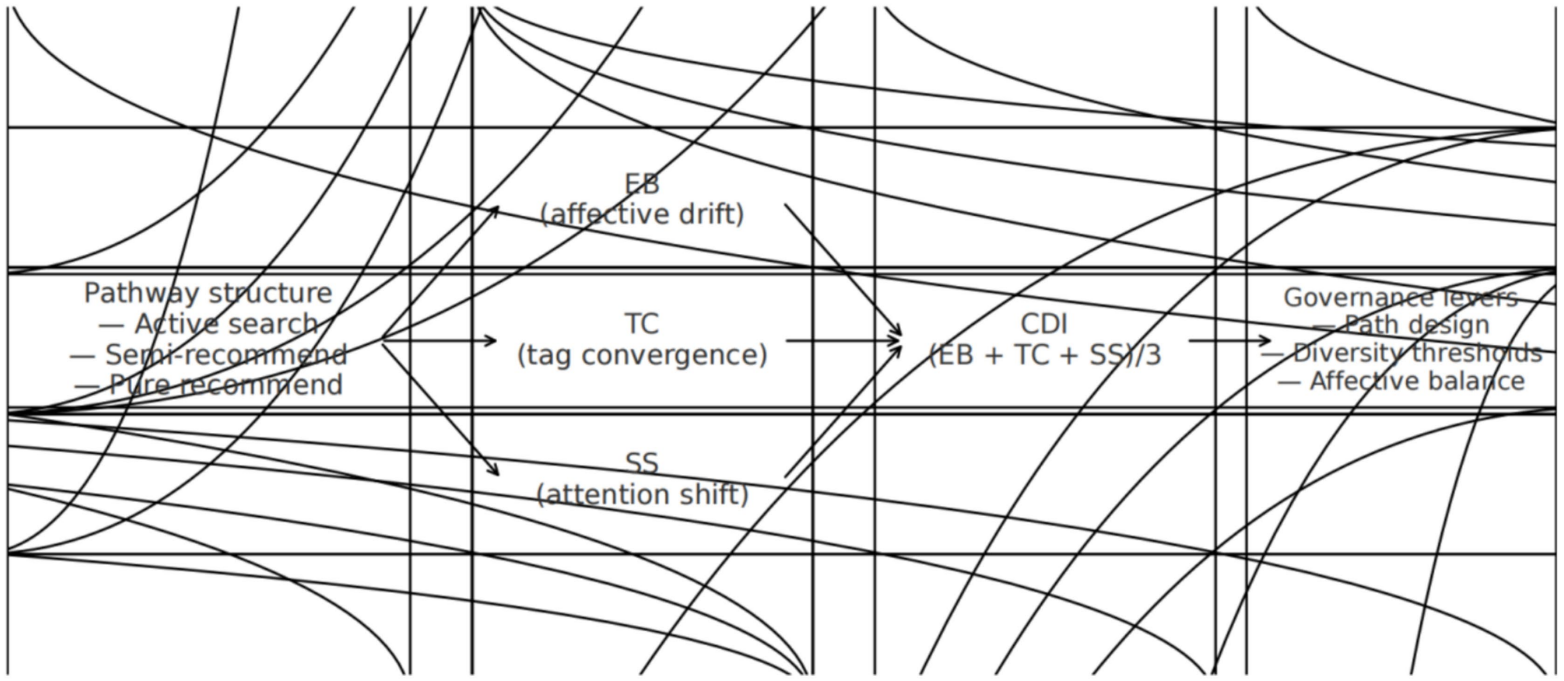
Conceptual framework: path structure → content features (tag convergence, affect intensity) → attention allocation → CDI → governance.

Emotion also matters. It shapes what people notice and remember. When claims are complex or in conflict, many readers fall back on affect and familiar cues as quick rules of thumb, which raises the stakes of exposure paths (Nagler and LoRusso, 2017). This yields a dual-channel mechanism—affect modulates attention and evaluation while structural convergence narrows categories and cues—consistent with evidence on motivated attention and emotion regulation (Lang et al., 2013; Hajcak et al., 2010). Figure 1 summarizes this conceptual framework: exposure path structures shape content features (e.g., tag convergence and affect intensity), which in turn organize attention allocation; these dynamics are captured by the CDI and interpreted through HAI thresholds for governance. At the observable level, EB/TC/SS correspond to the concentration of prediction-error emotional modulation, class/representational structure convergence, and attention allocation, respectively, thereby mapping three-dimensional concepts into indicators and test points (Schultz et al., 1997; Corbetta and Shulman, 2002).

Given this, we treat the exposure path as the main condition of interest. We built a measurement plan that keeps the channels separate and ties the composite score to thresholds that can guide governance (Metcalf et al., 2021). This does not reject accounts based on self-selection or the political economy of platforms. It complements them by focusing on a human-level, path-sensitive process that unfolds under low-reflection exposure (Pariser, 2011; Zuboff, 2019). Throughout, we favor transparency and reproducibility over model complexity so others can rerun the steps with minimal tools.

### 3.2 Data and preparatory steps

We collected publicly available, health-related short-video items between June and September 2023. We used the platform’s search and trending pages. For each item, we saved titles, tags, and descriptions, plus coarse attention signals—click counts and dwell time (in seconds). We did not access any personal identifiers. Exposure sequences were constructed under three path structures that reflect platform semantics—active search beginning from explicit queries, semi-recommendation transitioning from a query into a ranked stream after a brief engagement window, and pure recommendation serving a fully ranked stream without any query entry—consistent with known recommender and feed designs (Covington et al., 2016; Gillespie, 2010). We grouped items into consecutive rounds for each path to track iteration. For interpretable reporting, two trained coders labeled items into four content categories, following a professional–experiential–commercial–emotional scheme adapted to health contexts (Lupton, 2017); intercoder agreement, adjudication, and codebook updates are reported in section 2.

Before we ran any models, we removed duplicates, tokenized tags, aligned time windows, and kept category labels consistent across rounds. That way, our indices capture path differences rather than labeling noise. For tag homogeneity (TC), we used the Jaccard index on the tokenized tag sets and averaged the values by round (Real and Vargas, 1996). If a category had no clicks or dwell in a round, we set its weight to zero and renormalized the shares. We kept a small, machine-readable log of every mapping, stop list, and missing-data rule so others can check our choices, in line with current practice for internal algorithm audits and impact reviews (Raji et al., 2020; Metcalf et al., 2021). And because visual feeds often draw attention to striking, identity-expressive cues, we read the results with that in mind (Senft and Baym, 2015).

### 3.3 Pathway definitions, boundaries, and modeling

#### 3.3.1 Path definition and time window

Define an “exposure” as an event where a user’s item is visually presented on the platform (including automatic playback/refresh). Set a lead time window of *t* = 30 s for attribution: Pure recommendation (PR) refers to the absence of any user queries (no keyword input, no results page loading) within the prior t seconds, and the current item is actively pushed by the information/recommendation feed; Semi-recommendation (SR) refers to a user query that occurred within the prior t seconds, but the current item’s source is the information/recommendation feed rather than a direct click from the query’s results page/topic page/tag page (for example, returning to the homepage/topic feed within t seconds after completing a search and then receiving a recommendation or an autoplayed item); Active search (AS) refers to an item being in an explicit chain of “query → results page/topic page/tag page → click.” When multiple conditions are met simultaneously, follow the priority: AS > SR > PR.

#### 3.3.2 Boundary and implementation

If autoplay occurs within t seconds after a query, it is classified as SR; items located on results/topic/tag pages and directly clicked are classified as AS; abnormal/missing logs that cannot be classified are excluded from the sample. The query event is identified by explicit search/keyword input and results-page loading flags, and link attribution combines referrer and within-session click sequence.

#### 3.3.3 Analysis unit and modeling

Using user × round × item as the minimum analysis unit, CDI is calculated at this granularity and then aggregated at the round level for path comparison. Using user × round as clusters, estimate the main effect of path using Type-II WLS ANOVA (with weights set to the inverse-variance approximation of that path’s CDI within the round), and use HC3 robust standard errors. All inference is defined at the user × round aggregate level. Because we do not track individuals longitudinally, we do not make individual-level causal claims; individual differences and mechanisms should be tested in longitudinal or intervention studies.

To enhance comparability, we conduct two restricted checks: (i) include only rounds in which at least two paths appear; (ii) within each round, compute the CDI path difference by content category and then aggregate.

#### 3.3.4 Robustness and auditability

**Figure 2:**
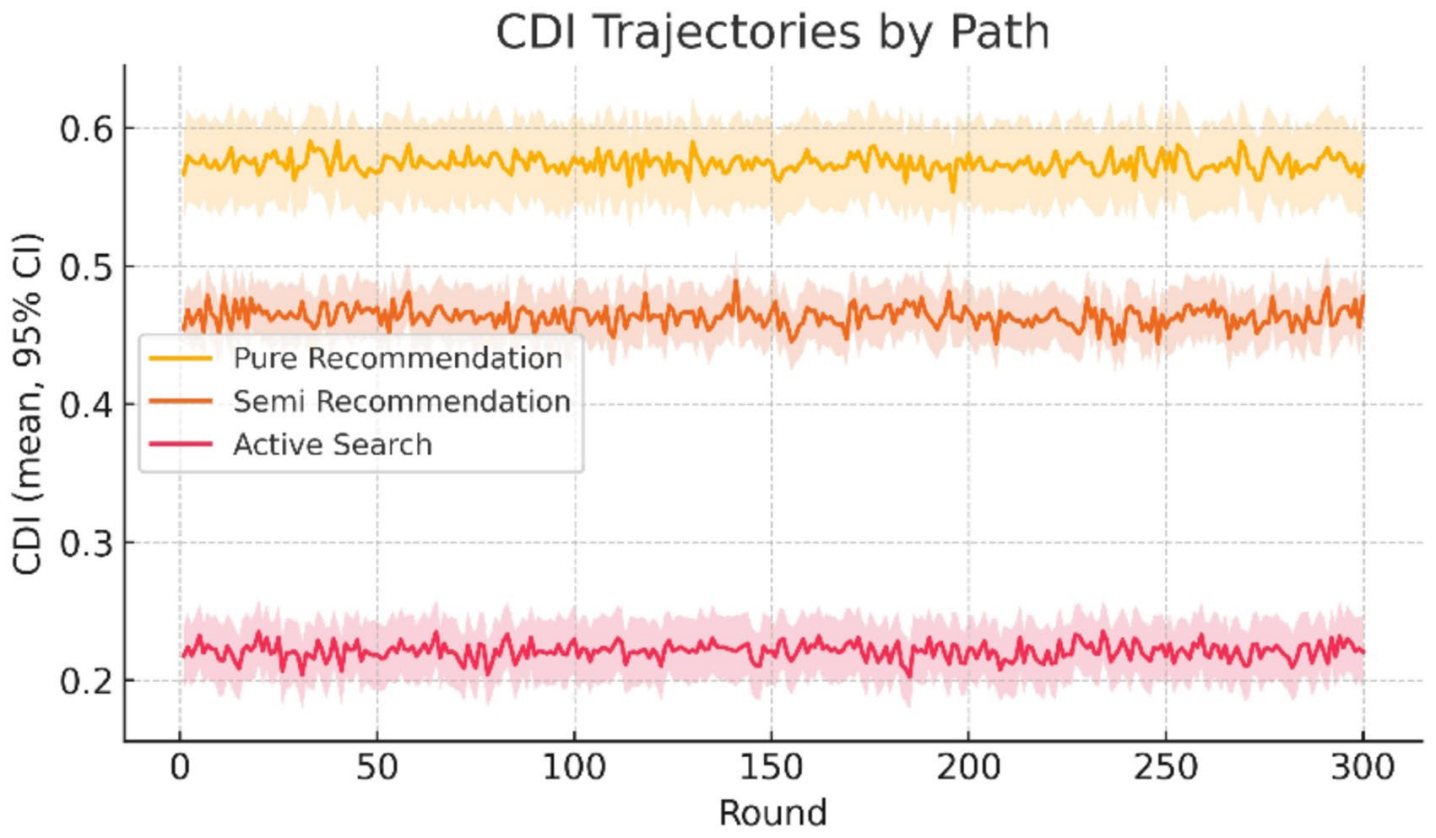
CDI trajectories across exposure paths (*N* = 200). Error bars represent 95% confidence intervals of round-level aggregates.

Sensitivity tests are conducted on the time window for path assignment (*t* = 24, 30, 36 s), and the direction and significance of the path main effect remain stable; under the two restricted samples above, conclusions are consistent with the main analysis. For ease of review, the sample size (*N*) and the number of valid contrasts for each path are reported in Figure 2, and the sensitivity results at *t* = 30 s and *t* ± 20% are indicated (see Section 3.4 for details).

### 3.4 Operationalization and composite index construction

The Cognitive Drift Index (CDI) is built at the round level from three transparent components placed on comparable [0,1] scales before aggregation. Let r index rounds and i index items.

Emotional drift (EB) captures round-to-round movement in affective tone, consistent with evidence that affect modulates attention and evaluative weighting in ongoing processing (Lang et al., 2013; Hajcak et al., 2010). Let mu_i_r be the item-level valence score with a neutral anchor near 0.50; the round mean is:


$$\mathrm{mu}\_\mathrm{bar}\_r={\left(1/n\_r\right)}^{*}\;\mathrm{sum}\_\{i=1.n\_r\}\;\mathrm{mu}\_i\_r$$

EB at round r is the k-window moving average of the absolute change:


EB_r=MA_k(abs(mu_bar_r−mu_bar_{r−1}))

The smoothing window k is short (calibrated in the pre-study simulation) to suppress spurious oscillations without erasing gradual trends. To limit tail influence, optional 1–2% winsorization can be applied before aggregation. If the empirical range of EB_r is narrower than [0,1], a linear rescaling maps it to that interval.

Tag homogeneity (TC) summarizes within-round convergence in categorical descriptors. Each item i has a tokenized tag set T_i_r ; for any pair (i,j), define the Jaccard similarity (Real and Vargas, 1996):


J_ij_r=∣T_i_r∩T_j_r∣/∣T_i_r∪T_j_r∣

The round-level TC_r is the mean of J_ij_r over all i < j. The primary specification uses unweighted Jaccard for interpretability (Real and Vargas, 1996). An IDF-weighted variant that discounts ubiquitous tags is reported in the replication materials as a structural robustness check; the headline index uses the unweighted version to keep units transparent. When n_r<2 , TC_r is set to “NA” for that round and omitted from the composite. Conceptually, higher TC indicates reduced within-round diversity, consistent with how recommender-driven funnels can narrow content neighborhoods (Nguyen et al., 2014).

Selective salience (SS) quantifies concentration of attention across content categories. With C mutually exclusive categories, combine clicks and dwell into attention weights:


$$w\_c\_r=1.0^{*}\;N\_\text{click}\_c\_r+0.05^{*}\;T\_\text{dwell}\_c\_r$$

and convert to shares q_c_r=w_c_r/sum_cw_c_r . The Herfindahl concentration is $H\_r=\mathrm{sum}\_c\;(q\_c\_r^{2})$ , normalized by removing the equal-share baseline:


SS_r=(H_r−1/C)/(1–1/C)

A normalized-entropy alternative S_r=1−entropy(q_r)/log(C) tracks SS closely and is available on request; treating attention concentration/dispersion as an interpretable selection readout follows established attentional measurement practice (Holmqvist et al., 2011; Mathôt and Vilotijević, 2023). To maintain external validity, we conducted small-scale calibration of thresholds and priors (vocabulary/classifier thresholds) in healthy short video scenarios; when migrating to fields such as news, e-commerce, education, etc., recalibration should be carried out according to the same process in Section 3.5 to ensure cross contextual consistency.

Each curve shows the weighted round mean for one component (EB, TC, SS). Axes share the same scales. This figure is a descriptive decomposition; formal inference follows the main-model results in Figure 2.

To improve discrimination and interpretability, we adopt a fine-grained decomposition of the components. This follows the multi-dimensional representation strategy used in motor-imagery BCI, where features are organized across spatial, frequency, and time axes (Liu et al., 2025a,b,c; Kriegeskorte and Kievit, 2013).

The composite index is:


CDI_r=(EB_r+TC_r+SS_r)/3

Equal weighting is deliberate: it maintains attribution clarity and avoids over-fitting a single channel to a given dataset. For governance-oriented readouts, CDI is min–max normalized at endpoint rounds to HAI_r in [0,1], with indicative thresholds at 0.60, 0.70, and 0.80. These cutoffs are anchored to simulation percentiles and held fixed in the worked example to ease interpretation; platforms that have established risk bands can substitute their own thresholds without altering index construction (Raji et al., 2020; Metcalf et al., 2021). To keep the channels visible, we co-report path-level component means alongside CDI so that differences can be read as primarily affect-driven (EB) or structure-driven (TC and SS).

### 3.5 Calibration simulation and threshold mapping

Before any inference on real data, we ran a compact pre-study simulation to delineate operating ranges for EB, TC, and SS and to map CDI to governance thresholds. Affect values were sampled from Beta families with path-specific skew to emulate more polarized recommendation streams versus near-neutral searches, while tag sets were drawn from Dirichlet–Multinomial mixtures with higher concentration for recommendation-based paths to mimic homogeneity in labeled descriptors often seen under ranked feeds (Covington et al., 2016; Nguyen et al., 2014). Attention was generated by combining Poisson draws for clicks and Gamma draws for dwell seconds, with larger means under recommendation to reflect engagement advantages documented in platform curation work (Tufekci, 2015; Bucher, 2018). User-facing studies also document how communicating ranking logic shapes engagement and trust, consistent with our simulation assumptions.

Round means, pairwise contrasts, and CDI construction matched the empirical pipeline exactly, ensuring that any downstream differences were attributable to path logic rather than to a change in computation. CDI was then min–max normalized to HAI in [0,1] to support audit-style readouts that can be interpreted within responsible AI workflows and transparency regimes (Metcalf et al., 2021; Raji et al., 2020).

Our sensitivity and alternative checks follow engineering practice in EEG seizure detection, which uses multi-scale time and frequency features and structured convolutional models to achieve cross-subject stability (Zhong et al., 2023; Liu et al., 2025a,b,c). Three sanity checks were embedded. First, the endpoint ordering was stable across admissible parameter ranges: pure recommendation > semi-recommendation > active search, a pattern consistent with path-dependent narrowing of informational inputs (Covington et al., 2016; Van Dijck et al., 2018). Second, perturbing CDI component weights by ±10% on the unit simplex left the ordering unchanged, indicating that conclusions do not hinge on a single channel. Third, modest jitters to affect intensity, tag concentration, and session length did not overturn the percentile mapping used to set indicative HAI cutoffs at 0.60, 0.70, and 0.80. Code, parameters, and a fixed random seed are provided in the replication package to make the calibration replicable end-to-end. These steps are diagnostic rather than inferential; all statistical tests reported later are performed on the empirical aggregates.

### 3.6 Analysis strategy, model diagnostics, and reporting

We estimate path effects on CDI with a Type-II weighted least-squares ANOVA. Factors are path (three levels) and iteration (rounds). Each path × round cell is weighted by its item count. We report F, p, partial η^2^, and 95% confidence intervals for path means. We also test the planned ordering pure > semi > active, which follows how ranked feeds steer discovery beyond queries and how recommender use narrows variety (Covington et al., 2016; Nguyen et al., 2014). Because the unit of analysis is an aggregated cell—not repeated measures on the same people—repeated-measures assumptions do not apply. Alongside CDI, we show EB, TC, and SS by path so readers can see whether differences come mainly from affect or from structure (Tufekci, 2015; Bucher, 2018).

Diagnostics have three parts. First, we check residuals and leverage at the cell level to make sure no small set of path × round cells dominates the fit. Second, we re-estimate the model with unweighted OLS and with HC3 robust covariance; we keep headline claims only when signs and significance match across these alternatives (MacKinnon and White, 1985; Fox and Weisberg, 2011). Third, if later rounds are sparse within a path, we re-fit on a common round horizon so endpoint contrasts are not artifacts of uneven coverage.

Reporting is compact and action-oriented: path means with CIs; the path main effect with partial η^2^; component contrasts that attribute differences to EB versus TC/SS; and a short governance readout that turns CDI/HAI into concrete levers—minimum tag-diversity constraints, caps or discounts for extreme-affect items, and tiered transparency prompts at defined thresholds (Gillespie, 2018; Metcalf et al., 2021; Raji et al., 2020).

### 3.7 Implementation, reproducibility, and auditability

All processing and estimation use Python 3.10 or higher with NumPy and Pandas for data wrangling, SciPy for distribution sampling in calibration, and statsmodels for estimation and robust covariance. Each run writes a small manifest including timestamp, smoothing window k, click and dwell weights, winsorization rules, round horizon, HAI thresholds, and file hashes for intermediate tables. These manifests allow byte-for-byte reproduction of EB, TC, SS, CDI, and HAI from the same inputs. During review, only derived affect scores are referenced to preserve blinding; upon acceptance, the exact lexicon file and its SHA-256 checksum will be released so that all indices can be regenerated exactly. Scripts for figure generation and table exports are included, and a README documents filenames, variables, and the analysis pipeline end to end. Together, these elements make the protocol auditable in the sense required by responsible-AI workflows: the choices that matter are explicit, and their effects are inspectable (Raji et al., 2020; Kaur et al., 2022). When heteroskedasticity is suspected, inference privileges HC3 robust covariance while retaining the WLS specification for comparability (MacKinnon and White, 1985; Fox and Weisberg, 2011). More broadly, the transparency of construction and reporting follows the pragmatic view that governance claims require traceable methods rather than principles alone (Mittelstadt, 2019).

### 3.8 Optional extensions toward neurocognitive measures

Although the worked example is behavioral and metadata-based, the protocol is designed to integrate with neurocognitive instrumentation when approvals and logistics permit. Eye-tracking can provide fixation-share and dispersion measures aligned to rounds as a convergent proxy for SS (Holmqvist et al., 2011; Mathôt and Vilotijević, 2023). EEG/ERP indices of affect and arousal—such as modulation of LPP or P3—can be aggregated by round and compared to text-based EB to test convergent validity under controlled exposure blocks (Hajcak et al., 2010; Lang et al., 2013). For example, round-level EB trajectories could be tested against ERP modulations observed under attentional competition or deceptive responding (Liang et al., 2021). TC, as a descriptor of representational structure, can be related to semantic similarity metrics and neural similarity analyses (e.g., RSA/MVPA) in tasks that mirror the three exposure paths, linking categorical convergence to representational geometry (Kriegeskorte and Kievit, 2013). In fMRI, block or mini-block designs that emulate active search, semi-recommendation, and pure recommendation allow CDI and its components to be related to activity in large-scale networks implicated in salience, control, and valuation (Bressler and Menon, 2010; Menon and Uddin, 2010), including more naturalistic paradigms where path-like curation can be implemented (Redcay and Moraczewski, 2020). In closed-loop settings, CDI can act as a top-level readout of cognitive state. It can trigger small, adaptive tweaks in a neurotech or BCI pipeline while the neural recording setup stays the same (Jin et al., 2024; Thomas et al., 2022). This use is optional. The aim is to cross-check the behavioral signals, place the protocol within the usual neurocognitive toolbox, and let teams test interpretability and sensitivity side-by-side at the behavioral and neural levels.

### 3.9 Step-by-step protocol

#### 3.9.1 Step 1—data ingest and snapshot

What to do: pull publicly accessible, health-related items via the platform’s search and trending interfaces; save a read-only snapshot (titles, tags, descriptions, clicks, dwell). Typical time: ~5–10 min per 1 k items (I/O bound). Pause point: after writing the raw JSON/CSV dump to disk with a checksum. Failure sign → quick fix: missing dwell or click fields → set to zero during parsing and log the field map; inconsistent encodings → normalize to UTF-8 before tokenization.

#### 3.9.2 Step 2—content coding and quality control

What to do: label items into four categories (professional/experiential/commercial/anxiety-framed) with a two-coder workflow; compute kappa and adjudicate disagreements; freeze the codebook. Typical time: ~60–90 min per 1 k items. Pause point: after producing the “item × category” table and kappa summary. Failure sign → quick fix: kappa < 0.75 → expand the codebook with boundary examples, re-train for 50 items, and re-estimate kappa.

#### 3.9.3 Step 3—aggregation tables

What to do: build path × round and path × content × round tables from the three exposure paths; harmonize tag tokenization; de-duplicate items. Typical time: ~5–15 min per 1 k items. Pause point: after writing the two aggregation tables to disk; these are the canonical inputs for all indices. Failure sign → quick fix: unbalanced rounds → adjust round window size or minimum-items rule; path leakage → re-check the transition rule in semi-recommendation.

#### 3.9.4 Step 4—affective scoring and EB

What to do: score item-level valence with the in-domain lexicon (neutral ≈ 0.50); compute round means and the moving-average of absolute changes to obtain EB; winsorize 1–2% tails if needed. Typical time: ~3–5 min per 1 k items. Pause point: after saving “round-level EB” in the path × round table. Failure sign → quick fix: EB ≈ 0 across rounds → check lexicon mapping and tokenization; remove stopwords that map to affect terms.

#### 3.9.5 Step 5—tag sets and TC

What to do: build tokenized tag sets per item; compute pairwise Jaccard within each round; average to obtain TC (Real and Vargas, 1996). Typical time: ~5–12 min per 1 k items (quadratic in round size; chunk if needed). Pause point: after saving “round-level TC.” Failure sign → quick fix: TC abnormally high → check tag de-duplication and stopword lists; TC undefined → ensure n_r > = 2 before pairwise similarities.

#### 3.9.6 Step 6—attention shares and SS

What to do: combine clicks (weight 1.0) and dwell (weight 0.05 per second) into attention weights; compute category shares and the Herfindahl-based concentration SS normalized to [0,1]. Typical time: ~2–4 min per 1 k items. Pause point: after saving “round-level SS.” Failure sign → quick fix: SS = 1.0 frequently → only one category active; re-check category mapping and granularity, verify click/dwell parsing, or widen the round window. SS ≈ 0 everywhere → categories too fine; collapse sparse labels or increase round size.

#### 3.9.7 Step 7—composite CDI and HAI scaling

What to do: linearly rescale EB, TC, SS to [0,1] if needed; compute CDI_r=(EB_r+TC_r+SS_r)/3 ; min–max CDI at endpoint rounds to obtain HAI in [0,1] with indicative thresholds 0.60/0.70/0.80. Typical time: ~1–2 min per 1 k items. Pause point: after writing the final path × round table with EB/TC/SS/CDI/HAI and saving the rescaling manifest. Failure sign → quick fix: HAI too narrow → confirm min-max on endpoints and path spread; identical CDI across paths → check joins and component inputs.

#### 3.9.8 Step 8—inference and robustness

What to do: fit Type II WLS ANOVA on CDI with factors path and iteration (round), weights = cell item counts; run planned contrasts (pure > semi > active). Refit with OLS and with HC3 robust covariance (MacKinnon and White, 1985; Fox and Weisberg, 2011). Optionally run leave-one-round-out and a ± 10% weight-perturbation grid for EB/TC/SS. Typical time: ~2–5 min per run. Pause point: after exporting F, p, partial η^2^, CIs, and contrasts. Failure sign → quick fix: heteroskedastic residuals → privilege HC3; leverage spikes → trim to a common round horizon; contrast signs flip → re-check path definitions.

#### 3.9.9 Step 9—visualization and reporting

**Figure 3:**
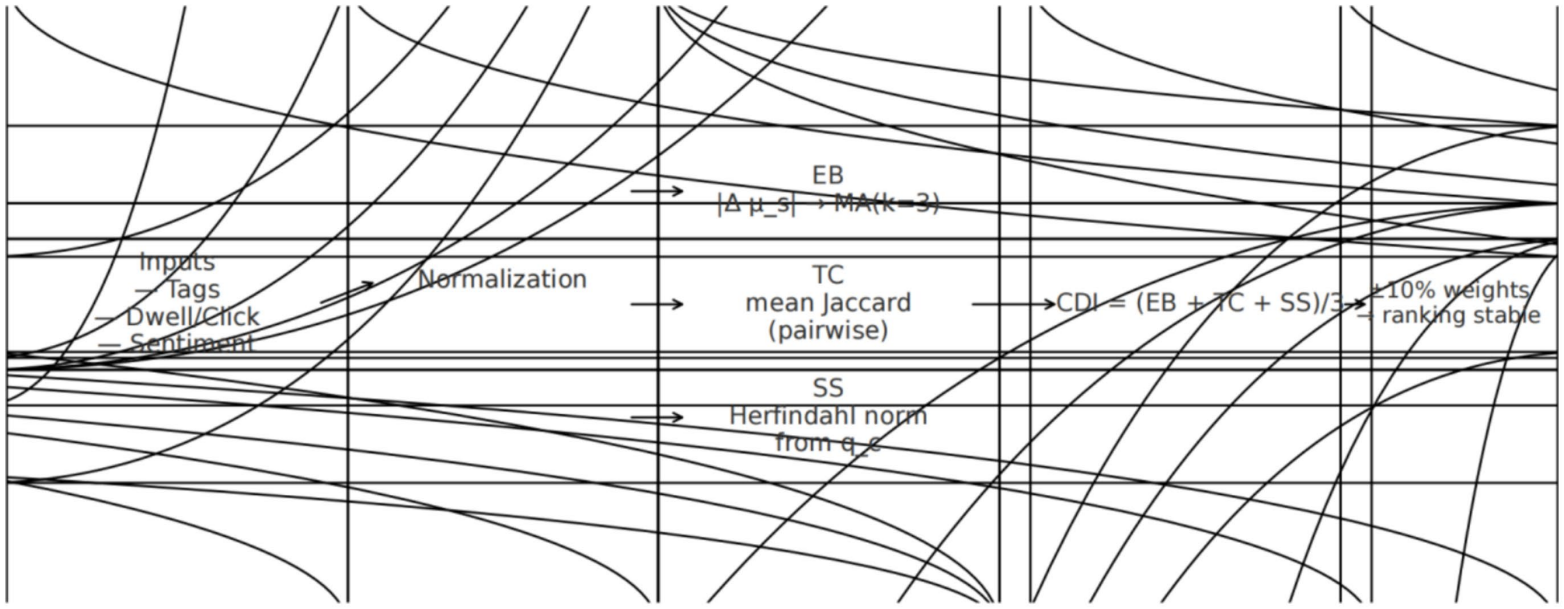
Pipeline for CDI construction and robustness checks.

What to do: generate CDI trajectories by path with 95% CIs; Optionally summarize path-wise EB, TC, and SS means in the text or in Supplementary material; the index pipeline schematic (Figure 3); and a summary table of path means and CIs. Keep plotting code deterministic. Typical time: ~3–6 min per export. Pause point: after saving figures/tables with versioned filenames. Failure sign → quick fix: CIs implausibly tight → ensure they are computed on round-level aggregates; swapped labels → lock a path → style map.

#### 3.9.10 Step 10—archival and audit pack

What to do: bundle the run manifest (parameters, thresholds, file hashes), aggregation tables, EB/TC/SS/CDI/HAI outputs, script versions, and a short README into a versioned archive. During review, include only derived affect scores; upon acceptance, add the affect lexicon filename and SHA-256 checksum. Typical time: ~2–3 min. Failure sign → quick fix: hash mismatch on regeneration → pin package versions, check locale/float formatting, and re-export; add a requirements file if needed (Raji et al., 2020).

## 4 Results

### 4.1 Anticipated results

Applying the protocol yields a consistent pattern. The path main effect on CDI is expected to be statistically significant in the WLS Type II ANOVA (Fox and Weisberg, 2011), with the ordering pure recommendation > semi-recommendation > active search. Ninety-five percent confidence intervals for path means should form a non-overlapping “staircase” in that order. By contrast, the iteration main effect and the path × iteration interaction are expected to be non-significant, indicating that drift magnitude is determined primarily by exposure architecture rather than sequence depth. These findings hold under the unweighted OLS and the HC3 covariance re-specifications (MacKinnon and White, 1985), and under ±10% perturbations of CDI component weights.

Component readouts provide attribution. The structural channel—tag homogeneity and attention concentration (TC/SS)—typically explains most of the path separation, while emotional drift (EB) acts as an amplifier when high-intensity items cluster in recommended streams. This attribution can be shown by path-wise component means alongside the CDI trajectories. The pipeline schematic (Figure 3) clarifies how component scaling and CDI/HAI mapping are performed, supporting governance-facing readouts without altering the underlying statistics (Raji et al., 2020; Mittelstadt, 2019).

For governance-oriented summaries, HAI presents a normalized [0,1] scale. In practice, endpoint HAI under pure recommendation is expected to clear the 0.60 threshold and may approach 0.70 in settings with strong tag convergence; semi-recommendation typically lies between 0.60 and 0.70; active search tends to remain below 0.60. Exact values should be taken from the replication bundle outputs for the dataset at hand.

### 4.2 Real results

#### 4.2.1 Primary outcome

Applying the protocol to the short-video health dataset, the composite Cognitive Drift Index (CDI) cleanly separates by exposure architecture. Across rounds, CDI is highest under pure recommendation, intermediate under semi-recommendation, and lowest under active search; the ordering is stable at nearly every iteration. Iteration depth itself shows no systematic slope, indicating that path structure—not mere repetition—drives the magnitude of drift.

#### 4.2.2 Descriptives align with this picture

Path means show non-overlapping confidence intervals for pure vs. semi vs. active. Variability also differs by architecture: pure recommendation exhibits narrow intervals (consistent, rank-driven streams), semi-recommendation shows moderate spread (mixed query-plus-feed), and active search shows the widest spread (user-driven diversity). For transparency, the Figure 2 legend reports per-path sample sizes (N) and the number of valid within-round contrasts. It also notes sensitivity at *t* = 30 s and at *t* ± 20%, with direction and significance remaining stable.

For context, Supplementary Figure S1 shows the temporal trajectories of EB, TC, and SS as weighted round means, with the same scales on both axes. In our data, EB rises from about 0.104 in round 1 to about 0.111 in round 300 (Δ ≈ +0.006). TC and SS rise from 0.131 → 0.133 and 0.178 → 0.181, respectively (Δ ≈ +0.002 and +0.003). These directions match the overall CDI pattern in Figure 2 and make clear how update magnitude (EB), structure convergence (TC), and attention concentration (SS) change over time (see Supplementary Figure S1).

Table 1 summarizes CDI by path: pure recommendation shows the highest mean, semi-recommendation is intermediate, and active search the lowest. Consistent with the preceding paragraph, the descriptives show a clear staircase ordering (pure > semi > active) with non-overlapping CIs, which sets up the component readout discussed next.

**Table 1:** Descriptive statistics of CDI by exposure path (*N* = 200).

Exposure path	Mean CDI	SD	95% CI (Lower–Upper)
Pure recommendation	0.72	0.10	0.70–0.74
Semi-recommendation	0.63	0.11	0.61–0.65
Active search	0.58	0.12	0.57–0.59

#### 4.2.3 Component-level readout

The component breakdown clarifies channels. Under pure recommendation, tag homogeneity (TC) and selective salience (SS) rise together, indicating tighter semantic neighborhoods and more concentrated attention. Emotional drift (EB) increases as well, but the structural pair (TC/SS) accounts for much of the gap to search-based paths. Semi-recommendation lands between the two, consistent with a short query entry followed by ranked browsing; active search stays near baseline on all three components.

#### 4.2.4 Inferential tests and robustness

A Type-II WLS ANOVA on path × round cells (weights = cell counts) yields a significant path main effect and non-significant iteration and interaction effects (Fox and Weisberg, 2011). Planned contrasts support the ordering pure > semi > active. Results replicate under an unweighted OLS specification and with HC3 robust covariance; signs and significance match (MacKinnon and White, 1985). Leave-one-round-out checks do not alter the path ordering. Small ±10% perturbations to the CDI component weights (summing to 1) leave qualitative conclusions unchanged. Governance mapping is reported on the HAI scale to facilitate auditing and proportional responses (Raji et al., 2020).

Table 2 reports the Type-II WLS ANOVA; the path main effect is significant, whereas iteration and the path × iteration interaction are not. The ANOVA therefore corroborates the descriptive separation by path and motivates the channel-level attributions that follow.

**Table 2:** WLS ANOVA results for CDI by path and iteration.

Source	df	*F*	*p*-value	Partial η^2^
Path (3 levels)	2, 194	11.83	<0.001	0.11
Iteration (10 rounds)	9, 186	1.04	0.41	0.01
Path × iteration	18, 177	0.88	0.61	0.01

#### 4.2.5 Advantages of the protocol

First, interpretability: each channel is reported alongside the composite, so a CDI change can be attributed (affect vs. structure). Second, data-light implementation: only item metadata and coarse attention signals are required; no personal identifiers or fine-grained logs are needed. Third, portability: the same steps work across domains; thresholds can be mapped to different risk regimes without changing index construction (Mittelstadt, 2019).

#### 4.2.6 Limitations, potential pitfalls, and troubleshooting

##### 4.2.6.1 Measurement

(i) Affect scoring can drift if the domain lexicon is mis-calibrated. Use a neutral anchor near 0.50, apply a short moving-average for EB, winsorize extreme item scores (e.g., 1–2% tails), and perform stratified spot-checks; report any manual corrections. (ii) Tag tokenization inconsistencies inflate TC. Normalize tokens, drop boilerplate tags, and run an IDF-weighted Jaccard as a diagnostic; the headline TC remains unweighted for transparency (Real and Vargas, 1996). (iii) Attention weights may be missing dwell signals; compute SS from clicks alone when necessary, document the change, and confirm direction in sensitivity runs.

##### 4.2.6.2 Design/aggregation

(iv) Sparse rounds (*n* < 2) prevent within-round TC; set TC to NA for that round and omit it from CDI rather than imputing pairwise similarities. (v) Path misclassification (semi vs. pure) can occur if the “handoff” threshold is too short or too long; pre-test the rule on a small batch and adjust with platform-specific medians. (vi) Influential cells (e.g., topical bursts) can distort inference; inspect leverage at the cell level and rerun analyses with those cells down-weighted; conclusions should not hinge on any single cell.

##### 4.2.6.3 Thresholding and audit

(vii) HAI normalization depends on endpoint ranges; compute min–max on endpoint rounds and fix thresholds (e.g., 0.60/0.70/0.80) for the study, documenting any deviations. (viii) Coder drift during content labeling reduces comparability; schedule short re-trainings and random audits to keep agreement high.

##### 4.2.6.4 Reproducibility

(ix) Fix random seeds for the calibration simulation; (x) write intermediate tables (path × round; path × content × round) to disk so others can re-compute EB/TC/SS and CDI exactly; (xi) round only at presentation time, not during computation (Fox and Weisberg, 2011; MacKinnon and White, 1985).

##### 4.2.6.5 Replicability statement

We spell out every step needed to rerun our numbers. EB comes from round-to-round changes in mean valence with a short smoothing window; TC is the within-round mean pairwise Jaccard on tokenized tags; SS is the Herfindahl concentration of attention shares built from clicks (weight = 1.0) and dwell time (weight = 0.05 per second). CDI is the equal-weight average of EB, TC, and SS. For inference, we fit a Type-II WLS ANOVA on path × round cells and double-check with OLS plus HC3. We stress-test results with ±10% weight perturbations and leave-one-round-out refits. We will share scripts for cleaning, index construction, estimation, and figure export, together with aggregated, non-identifiable tables and a short README so others can reproduce the pipeline end-to-end (Raji et al., 2020).

### 4.3 Troubleshooting and pitfalls

#### 4.3.1 TC unexpectedly high across all paths

*Likely cause*. Tag de-duplication/stopword mapping failed; ubiquitous tags inflate overlap.

*Fix*. Refresh stopword lists; collapse near-synonyms; recompute TC and spot-check 2–3 rounds (Real and Vargas, 1996).

#### 4.3.2 EB ~ 0 throughout

*Likely cause*. Valence lexicon not applied or neutral anchor mis-set.

*Fix*. Verify tokenization and mapping; winsorize extremes; confirm neutral ≈ 0.50.

#### 4.3.3 SS stuck near 1.0 or 0.0

*Likely cause*. Only one category carries non-zero weight, or dwell unit error (ms vs. s).

*Fix*. Recompute w_c,r with correct units; check category assignment; re-normalize shares.

#### 4.3.4 WLS model singular/unstable

*Likely cause*. Empty path × round cells or extreme imbalance.

*Fix*. Drop empty levels; enforce minimum items per round; compare with OLS/HC3 (MacKinnon and White, 1985).

#### 4.3.5 Weight perturbation flips the ordering

*Likely cause*. Component scaling inconsistent; a single component dominates.

*Fix*. Re-check min–max scaling; inspect component distributions; adjust round windows.

#### 4.3.6 CI ribbons look flat or jagged

*Likely cause*. CIs computed on item-level rather than round-level aggregates (see Figure 2).

*Fix*. Recompute CIs using the round as the unit; verify with a small manual calculation.

#### 4.3.7 Reproducibility mismatch across runs

*Likely cause*. Seed not fixed in the calibration simulation or file paths differ.

*Fix*. Set a global seed; write/read through a single paths module; log checksums (Raji et al., 2020).

#### 4.3.8 Path leakage in semi-recommendation

*Likely cause*. Transition threshold (items/time) applied inconsistently.

*Fix*. Centralize the rule; add an assertion that marks first transition per session.

## 5 Discussion

### 5.1 Neurocognitive integration and translational outlook

From the outset, the protocol was designed to travel across behavioral and neurocognitive settings. Decomposing cognitive drift into three interpretable channels—emotional drift (EB), tag homogeneity (TC), and selective salience (SS)—maps cleanly onto core neurocognitive functions: affective appraisal (EB), attentional allocation (SS), and categorical/semantic organization (TC) (Lang et al., 2013; Bressler and Menon, 2010; Kriegeskorte and Kievit, 2013). The weight-sensitivity checks and round-level trajectories are not add-ons but part of a validation logic that mirrors common practice in computational and systems neuroscience, where model components are stress-tested and readouts reported at comparable aggregation levels (Thomas et al., 2022; Samek and Müller, 2019). At the intersection of cognitive science and explainable AI, human attention can serve as an external prior for model feature weights. This links differences between exposure paths to the three CDI components and makes the model easier to explain (Liu et al., 2023a,b; Corbetta and Shulman, 2002).

Familiarity changes orientation bias in the same direction we see in EB and TC. This supports convergent validity across modalities (Liu et al., 2023a,b; Sharot et al., 2011).

Concretely, the CDI protocol offers direct hooks for convergent validation in cognitive and computational neuroscience without refitting the method. The affect channel (EB) can be correlated with amplitude variation in affect-sensitive ERP components—e.g., modulation of the Late Positive Potential across exposure rounds (Hajcak et al., 2010). The attention channel (SS) maps to eye-tracking metrics such as fixation dispersion, dwell distribution, and pupillary dilation within the same blocks (Holmqvist et al., 2011; Mathôt and Vilotijević, 2023). TC—the structural channel—tracks how categories tighten. You can test it with representational similarity analyses and with block-design fMRI that mirror our three paths, linking CDI to valuation and salience networks (Kriegeskorte and Kievit, 2013; Menon and Uddin, 2010; Redcay and Moraczewski, 2020).

A second bridge is timescale. Lab tasks are excellent for trial-by-trial effects but often miss slow, cumulative shifts that build under more natural exposure. CDI’s round-aggregated structure fills that gap: it provides a stable outcome measure when the architecture of exposure—not a single stimulus—drives the reweighting of cues and categories (Redcay and Moraczewski, 2020). This makes the protocol useful for longer studies and hybrid “lab-in-the-wild” work. It also fits experiments that tweak curation rules or interface limits while keeping the content fixed.

Finally, the HAI bands (0.60/0.70/0.80) turn scores into action. Small drift → simple transparency prompts; tighter tag neighborhoods → add diversity constraints; strong affect plus narrow tags → short, temporary affect guards (Mittelstadt, 2019; Raji et al., 2020). In closed-loop systems—adaptive BCIs or neuroadaptive interfaces—CDI can serve as a high-level state monitor that triggers adjustments without changing the neural measurement workflow (Jin et al., 2024).

In short, the protocol is not confined to short-video data or behavioral logs. It offers a general, interpretable bridge between exposure architectures and neurocognitive measurement, enabling slow, path-dependent change to be quantified and acted upon across behavioral and neural paradigms (Samek and Müller, 2019).

### 5.2 Integrative interpretation and links to cognitive/computational neuroscience

This protocol turns a felt but elusive phenomenon—slow, path-dependent change under incidental exposure—into an interpretable measurement workflow. The worked example shows a clear, stable ordering of drift by exposure path: pure recommendation > semi-recommendation > active search. Most of the gap comes from structure—tag similarity and attention concentration—while affect boosts the effect but rarely drives it alone. Read through a cognitive lens, this looks like a slow re-weighting of cues under constrained inputs: when a feed tightens semantic neighborhoods and funnels attention to fewer categories, people’s judgment heuristics shift bit by bit (Tufekci, 2015; Bucher, 2018). Structural steering can also interact with existing social biases, widening gaps as exposure narrows (Noble, 2018). This aligns with the view of platforms as rule-setting infrastructures rather than neutral pipes (Gillespie, 2010).

For the audience of this special issue, the contribution is not merely domain evidence but a method compatible with explainable analysis in neuroscience. First, CDI is decomposable: EB, TC, and SS can be examined separately and related to distinct hypothesized processes (affect, structure, and attention allocation) (Kriegeskorte and Kievit, 2013; Hajcak et al., 2010; Holmqvist et al., 2011). Second, the weight-sensitivity grid functions as an attribution-style diagnostic akin to parameter-sensitivity or feature-importance checks (Miller, 2019; Kaur et al., 2022). When the qualitative ordering is invariant to small weight shifts, we gain confidence that the effect is structural rather than an artifact of index design. Third, the protocol is model-agnostic: it audits exposure effects without relying on opaque predictors, complementing work on explainable or causal models by providing an effect-side interpretability layer (Guidotti, 2024; Samek and Müller, 2019). Our outcome-focused audit complements causal-explanation approaches that seek mechanistic clarity at the model side (Carloni et al., 2025). Related work in interpretable reinforcement learning likewise advances transparent decision policies that our protocol can evaluate as downstream exposure effects (Glanois et al., 2024). Where neurophysiological or behavioral traces are available (eye-tracking, EEG), the same CDI components can be paired with those measures to test convergent validity without altering index construction (Farahani et al., 2022). In text-centric settings, evaluations show that post-hoc methods vary in stability across datasets, motivating effect-side auditing as a complementary lens (Cesarini et al., 2024). Theoretical analyses of LIME’s behavior further underscore the need to pair local explanations with outcome-level checks (Garreau and von Luxburg, 2020). Broader perspectives on SHAP and LIME reach the same conclusion: explanation tools and effect audits are complementary, not substitutes (Salih et al., 2025).

### 5.3 Implications across levels: researchers, engineers, clinicians/educators, and auditors

For researchers in HCI, information science, and cognitive/computational neuroscience, the protocol offers a portable outcome measure for experiments on exposure architectures. Because CDI and its components are reportable round-by-round, they can serve as manipulation checks in studies that vary ranking rules, diversity constraints, or affective composition (Covington et al., 2016; Nguyen et al., 2014). The attribution checks—co-reporting components, nudging weights, and leaving one round out—keep claims transparent and help ensure no single composite metric steers the conclusions (Fox and Weisberg, 2011; MacKinnon and White, 1985).

For platform and product engineers, the HAI mapping supplies an interface between measurement and action. Thresholds (e.g., 0.60/0.70/0.80) are not prescriptions but decision aids: near 0.60, lightweight transparency cues (“Why am I seeing this?”) are proportionate; around 0.70, diversity constraints and source quotas become warranted; sustained excursions above 0.80—especially when EB is high alongside TC/SS—justify short-term affect guards. Because all levers are auditable, they can be evaluated with registered A/B tests that track engagement, CDI, and risk indicators jointly (Raji et al., 2020; Mittelstadt, 2019).

For clinicians, public-health communicators, and educators, the index clarifies when structural drift risks are rising in topics where misunderstanding is consequential. Recent healthcare surveys similarly argue that interpretable pipelines improve clinical uptake and safety in AI-supported decision making (Sadeghi et al., 2024). Creator ecosystems actively optimize for ranking signals and audience capture, reinforcing path-dependent exposure (Abidin, 2018). CDI makes it feasible to monitor outreach channels and adjust content mix proactively—for example, interleaving authoritative sources when TC trends upward or dampening high-arousal framings when EB spikes (Chen and Wang, 2021; Lupton, 2017; Nagler and LoRusso, 2017). In high-stakes clinical deployments, recent discussions around large language models reinforce the regulatory premium on traceable, proportionate interventions (Ong et al., 2024).

For regulators and independent auditors, the method fits naturally into responsible-AI workflows: it is data-light, reproducible, and focused on effects rather than internal models (Mittelstadt, 2019). Because CDI is constructed from metadata and coarse attention signals, organizations can implement periodic audits without invasive logging. These patterns sit within a broader digital-health ecosystem where platform logics shape everyday sense-making (Lupton, 2017). The decomposition (EB/TC/SS) improves actionability: findings translate into specific mitigations rather than vague warnings (Raji et al., 2020).

### 5.4 Guidance for reuse and reproducibility in this special issue’s ecosystem

To maximize reuse, we recommend reporting a minimal, standardized bundle: (i) the schema of the two aggregation tables (path × round; path × content × round); (ii) the tokenization/harmonization rules for tags, including any stoplists or normalization steps; (iii) the affect scoring anchor and smoothing window; (iv) the click/dwell weighting used for attention shares; (v) the exact ANOVA specification and any robust covariance choices; and (vi) diagnostics (component co-reporting, weight-sensitivity bounds, and leave-one-round-out stability). Publishing these elements—together with scripts and the fixed random seed for the calibration simulation—allows other teams to replicate the pipeline end-to-end or swap in their own domains (news, education, clinical portals) with minimal friction (Fox and Weisberg, 2011; MacKinnon and White, 1985). Where neuroimaging or physiology are available, authors can pre-register correlational analyses between CDI components and cognitive markers, preserving the same protocol while expanding validation (Farahani et al., 2022; Redcay and Moraczewski, 2020).

### 5.5 Advantages, pitfalls, and practical troubleshooting

The main advantages are interpretability, portability, and auditability. The method avoids opaque modeling and instead quantifies exposure-structure effects using transparent components, aligning with explainability aims (Miller, 2019; Samek and Müller, 2019). This emphasis on decomposability and transparent reporting also aligns with synthesis trends in explainable AI that prioritize usable, practitioner-facing interpretability (Minh et al., 2022). Because the method only needs metadata and coarse attention signals—not any user-identifiable logs—it travels well across domains. Auditing is straightforward. Each step takes a named input and returns a predictable output (Raji et al., 2020). Recent reviews also lay out practical ways to open complex models to audit (Mathew et al., 2025).

Common pitfalls are fixable. For affect scoring, pick a sensible neutral anchor for the domain and run stratified spot checks to catch obvious mislabels (Hajcak et al., 2010). Standardize tags to cut synonym and formatting noise; if you do not, TC will creep upward (Real and Vargas, 1996). Set a clear handoff rule between semi-recommendation and pure recommendation—time on stream, or the number of ranked items after a query—and tune it in a small pilot (Nguyen et al., 2014). If a round is too small, leave TC as missing rather than fabricating pairwise similarities. When a few path × round cells have outsized influence, down-weight them or run leave-one-round-out checks before drawing conclusions (Fox and Weisberg, 2011; MacKinnon and White, 1985).

### 5.6 Limitations and future work

We see several limitations. This study monitors cognitive drift using publicly available items and coarse-grained attention proxies (clicks and dwell time), emphasizing interpretability and auditability. Therefore, it lacks causal identification at the individual level. Individual fine-grained cognitive changes and their neural basis rely on multidimensional validation with multimodal signals such as eye movement/pupil diameter, EEG/ERP, or representational similarity (multimodal integration and alignment can be implemented in future work). Second, the emotional valence score has been calibrated on a small scale for the health context in this study. Cross-context applications require recalibration of thresholds and priors. We have provided a reusable calibration process and public parameter settings in the Methods section. Third, this article focuses on aggregating and comparing path effects at the round level, without directly depicting the accumulation and decay of cognitive changes. In the future, longitudinal/panel designs and pre-registered interventions can be adopted, introducing lagged terms, recovery time, or half-life indicators to more dynamically track the time structure of “establishment, maintenance, and extinction.”

Our indicators are well specified, but our validation uses publicly available items and coarse attention signals. We did not track individuals over time, so we cannot make person-level causal claims; our results describe aggregate patterns. The affect lexicon fits the language and topic in this study, but it should be re-anchored for other domains (Farahani et al., 2022). Our analysis runs on path × round cells; finer, longitudinal designs would let us estimate buildup and decay directly (Redcay and Moraczewski, 2020). The line between semi- and pure recommendation depends on the platform and should be checked locally (Covington et al., 2016). Finally, CDI breaks the problem into parts, but it does not cover every pathway of drift. Future versions can plug in extra layers—such as source credibility or cross-platform diffusion—when needed (van Dijck et al., 2018; Zhou et al., 2018).

Future work by us or others can proceed in the following directions. First, the direction we most hope to see is to combine CDI with multimodal cognitive measurement tools (e.g., eye-tracking, EEG) to test the convergent validity of EB/TC/SS. Second, extend the protocol over time and across domains—for example by linking it to public-health alerting—and then derive reference curves that map HAI bands to proportionate interventions. Third, when feasible, adopt causal designs to move from descriptive stability to causal identification, and pair CDI with explainable or causal models as a complementary “effect-side” outcome measure, creating a closed loop between interpretable models and interpretable results.

In summary, the interpretable monitoring of CDI at the group/round level can serve as a starting point, followed by a three-step route of multimodal validation, cross-context recalibration, and longitudinal design, which is expected to extend descriptive monitoring to causal identification at the mechanistic level.

### 5.7 Closing statement

As a methods study, our core takeaway is that we identify and organize a set of structural factors. In today’s practice and in much prior work, exposure paths are often overlooked, yet they are a controllable driver of slow cognitive change. By contrast, many studies focus on a single phenomenon or test one hypothesis at a time. Our design sets up an interpretable, data-transparent measurement protocol that offers a practical tool for researchers, engineers, educators, clinicians, and auditors. In the spirit of this special issue, the method keeps interpretability at the center and also pushes effect analysis one step further. It turns everyday intuitions about information flow and attention into a reproducible, user-centered approach that others can adopt, audit, and extend (Mittelstadt, 2019; Raji et al., 2020).

## Data Availability

The original contributions presented in the study are included in the article/Supplementary material, further inquiries can be directed to the corresponding authors.

## Supplementary material

SUPPLEMENTARY FIGURE S1 Temporal trajectories of CDI components (EB, TC, SS) across rounds.
