# Supplementary figures and images for: Auditing cognitive drift in AI-driven recommendation: a responsible AI methods protocol with a health case demonstration
Source: Front Neurosci. 2025 Dec 8;19:1697053. doi: 10.3389/fnins.2025.1697053 (PMC12722931; doi:10.3389/fnins.2025.1697053)

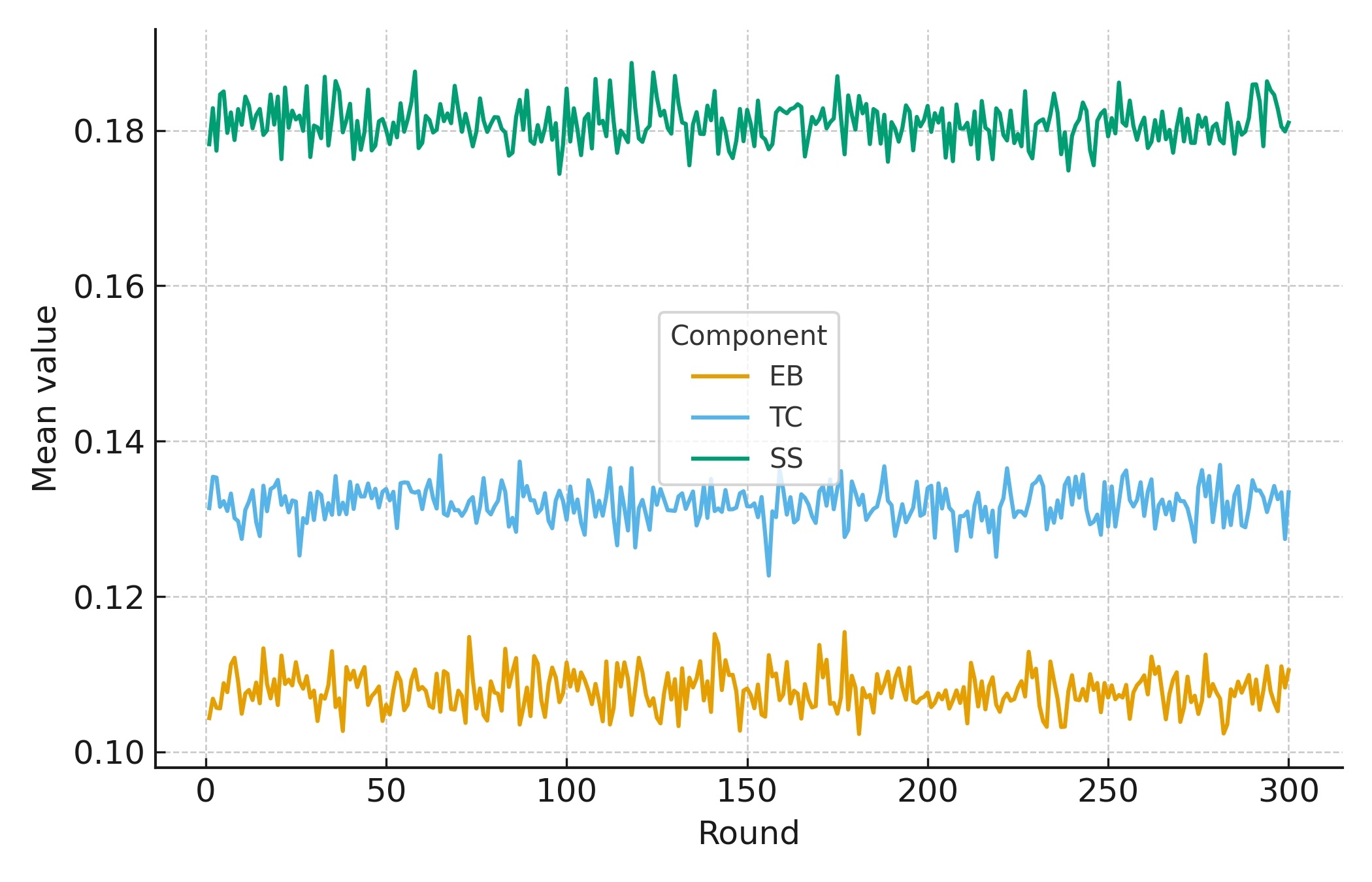

Supplement: SUPPLEMENTARY FIGURE S1 — Temporal trajectories of CDI components (EB, TC, SS) across rounds. [file Image_1.jpeg]
